# Expression of Scytonemin Biosynthesis Genes under Alternative Stress Conditions in the Cyanobacterium *Nostoc punctiforme*

**DOI:** 10.3390/microorganisms10020427

**Published:** 2022-02-12

**Authors:** Janine Bennett, Tanya Soule

**Affiliations:** Department of Biological Sciences, Purdue University Fort Wayne, Fort Wayne, IN 46805, USA; Janine.Bennett@osumc.edu

**Keywords:** scytonemin, ultraviolet radiation, high light, cyanobacteria

## Abstract

The indole-alkaloid scytonemin is a sunscreen pigment that is widely produced among cyanobacteria as an ultraviolet radiation (UVR) survival strategy. Scytonemin biosynthesis is encoded by two gene clusters that are known to be induced by long-wavelength radiation (UVA). Previous studies have characterized the transcriptome of cyanobacteria in response to a wide range of conditions, but the effect on the expression of scytonemin biosynthesis genes has not been specifically targeted. Therefore, the aim of this study is to determine the variable response of scytonemin biosynthesis genes to a variety of environmental conditions. Cells were acclimated to white light before supplementation with UVA, UVB, high light, or osmotic stress for 48 h. The presence of scytonemin was determined by absorbance spectroscopy and gene expression of representative scytonemin biosynthesis genes was measured using quantitative PCR. Scytonemin genes were up-regulated in UVA, UVB, and high light, although the scytonemin pigment was not detected under high light. There was no scytonemin or upregulation of these genes under osmotic stress. The lack of pigment production under high light, despite increased gene expression, suggests a time-dependent delay for pigment production or additional mechanisms or genes that may be involved in scytonemin production beyond those currently known.

## 1. Introduction

As phototrophic organisms, cyanobacteria must live in environments that are regularly exposed to solar ultraviolet radiation (UVR) [1]. Ultraviolet A radiation (UVA; 320–400 nm) is a major contributor to cell damage from solar energy [2] since more than 95% of solar UVR that reaches the Earth’s surface is within the UVA range. The remainder of the UVR that reaches Earth comes from ultraviolet B radiation (UVB; 280–320 nm) [3,4]. UVA indirectly damages DNA by interaction with cellular chromophores, which lead to the production of DNA-damaging reactive oxygen species. These reactive oxygen species also go on to damage other biomolecules, resulting in UVA as a major contributor to cell damage [2,5,6,7,8]. 

Cyanobacteria have evolved several defense strategies against UVA radiation [1,9,10,11]. One strategy employed is the production of the indole-alkaloid sunscreen pigment, scytonemin. When scytonemin accumulates extracellularly following UVR exposure, it intercepts harmful photons before they damage cellular machinery. Scytonemin remains stable in the presence of abiotic stressors and is reported to block up to 90% of UVR from entering the cell [12,13]. In addition to its protective ability against UVR, scytonemin shows antioxidant capabilities and nontoxicity [14,15,16,17].

**Figure 1:**
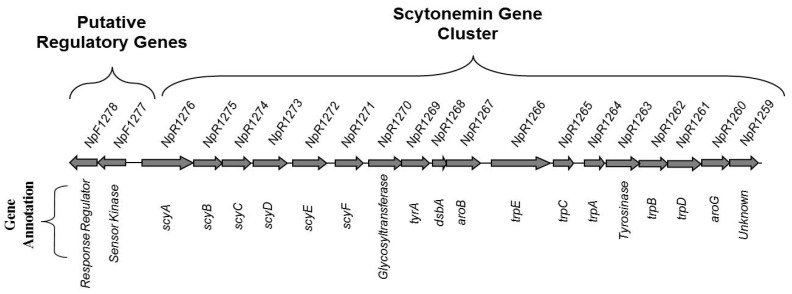
Main scytonemin biosynthetic gene cluster and regulatory genes in the *N. punctiforme* genome.

Scytonemin biosynthesis is encoded by two conserved gene clusters in the cyanobacterium *Nostoc punctiforme*. The main biosynthetic cluster includes 18 genes (Npun_R1276 to Npun_R1259) regulated by an upstream two-component regulatory system (TCRS; Npun_F1278 and Npun_F1277) (Figure 1) [18,19,20,21]. Interestingly, the sensor kinase, Npun_F1277, contains PAS/PAC domains [22]. In other studies, these domains have been shown to bind to small molecules in response to stimuli, such as light, oxygen, pH, or salinity [23]. The other cluster of five genes (NpunF_5232 to NpunF_5236), known as the *ebo* cluster, is involved in precursor transport across the periplasm [18,24]. The downstream genes in the main gene cluster are involved in synthesizing amino acids, which are then utilized by the upstream genes *scyA* to *scyC* (Npun_R1276 to Npun_R1274) to synthesize specific scytonemin precursors [25,26,27]. Both gene clusters and the TCRS are highly conserved among closely related species of cyanobacteria, with the *ebo* cluster also occurring among diverse bacteria and algae [18,24].

Although UVA alone can elicit the biosynthetic response, other studies have demonstrated that scytonemin induction can be enhanced by UVA supplemented with UVB, high light, and osmotic stress [13,28,29]. While these studies identified alternative stresses that can induce scytonemin biosynthesis, some were conducted in conjunction with UVA, while none of them were conducted at the gene expression level or in *Nostoc punctiforme*.

The objectives of this study are to evaluate the presence of scytonemin in *Nostoc punctiforme* and measure the transcriptional response of scytonemin biosynthetic genes in response to UVA, UVB, high light, and osmotic stress. It is widely accepted that UVA induces the production of scytonemin and a previous study revealed a synergistic or antagonistic effect, when cells were stressed with other environmental factors in conjunction with UVA [12,28]. Furthermore, expression of the main scytonemin biosynthetic gene cluster, the TCRS, and the *ebo* cluster have all been shown to increase in response to UVA stress [30,31]. Additionally, under short-term UVB and high light stress (20 to 60 min), the TCRS responded with an increase in gene expression [31]. Given these previous studies, it is expected that the light-associated environmental conditions (UVA, UVB, and high light) will induce the expression of genes from both gene clusters, while a non-light-associated condition, such as osmotic stress will downregulate these genes. To test this hypothesis, the expression of representative genes from each gene cluster were measured using quantitative-PCR following stress exposure. Genes *scyA* and *trpB* were measured to represent the main biosynthetic cluster and *eboE* was measured for the *ebo* gene cluster. A previous study showed that the main scytonemin gene cluster is transcribed as two main transcripts, the first starting with *scyA* and the downstream transcript starting with *trpE* [30]. Therefore, *scyA* and *trpB* were chosen to record expression levels from each transcript. The *eboE* gene (Npun_F5233) is the fourth gene in the cluster. It was selected since it showed the strongest expression in response to UVA out of any of the *ebo* genes in a previous study [30].

This study adds value to the previous body of research since it directly connects various elements of what we know regarding scytonemin biosynthesis and how it relates to environmental conditions. For instance, there are studies that have focused on the induction of scytonemin under different conditions [28], others that have looked at the whole transcriptomic response to these conditions [32,33], and still others that connected scytonemin gene expression to only UVA stress [30]. To date, no single study has targeted the intersection of scytonemin biosynthesis, the associated gene expression response, and compared them under various conditions. Furthermore, any effort to mass produce scytonemin in a native host will need a thorough understanding of the regulatory mechanisms governing its synthesis to maximize efficiency. Overall, by identifying alternative ways to induce scytonemin at the gene expression level, this study will help us better understand the various ways that scytonemin may play a role in the cyanobacterial response to diverse environmental conditions.

## 2. Materials and Methods

### 2.1. Strain, Culture Conditions, and Pigment Extraction

Cultures of *Nostoc punctiforme* ATCC 29133 (PCC 73102; a gift from Ferran Garcia-Pichel) were grown under white light in sterile flasks containing the Allen and Arnon (AA/4) growth medium [34]. For high light and UVR exposure, *N. punctiforme* cells were filtered onto 90 mm polycarbonate membrane filters and the filters were placed floating on a nitrogen-free AA/4 medium in sterile glass dishes to allow for an even illumination and growth. All of the cultures remained under white light to acclimate and allow for initial growth. The conditions used for scytonemin induction studies were adapted from Dillon, Tatsumi, Tandingan, and Castenholz [28] and Soule, Garcia-Pichel, and Stout [30]: White light (~40 µmols photons m^−2^ s^−1^) was supplemented with UVA (5 W m^−2^; continuous), UVB (0.5 W m^−2^; 3 h per day), or osmotic stress (5 g NaCl L^−1^; continuous) and high light was provided at a final intensity of 100 µmols photons m^−2^ s^−1^ (continuous). For UVB stress, transparent glass lids were replaced with a plastic film for optimal UVB penetration. Each stress condition was provided for 48 h since previous data showed that expression of the scytonemin biosynthetic genes peak after 48 h of continuous UVA exposure [30]. Control cultures for each experimental variable were grown under standard (white light) conditions in the absence of the stress. After the stress period, cells were vortexed off the filter into a sterile AA/4 medium and then concentrated by centrifugation, frozen in liquid nitrogen, and stored at −80 °C until RNA processing.

To assess scytonemin pigment production following exposure, cells were opened by grinding with a glass mortar and pestle and scytonemin was extracted in 100% acetone in the dark overnight. The presence of scytonemin was distinguished from other pigments by its ability to absorb light at 384 nm [12].

### 2.2. Expression of Scytonemin-Associated Genes

Total RNA was isolated from the cells using bead-beating and LiCl precipitation [35]. DNA was degraded in each sample with the Ambion TURBO DNA-free™ Kit (Life Technologies), according to the manufacturer’s protocol. DNase-treated RNA (2 µg) was converted to cDNA using the Sensi-Fast cDNA Synthesis Kit (Bioline). Transcript levels of the scytonemin-associated genes, *scyA* (Npun_R1276), *trpB* (Npun_R1262), and *eboE* (Npun_F5233) were measured in triplicate with quantitative-PCR on a CFX Connect Real-Time PCR System (Bio-Rad Laboratories) using the iScriptTM Reverse Transcription Supermix for RT-PCR (Bio-Rad Laboratories) and primers from Soule, Garcia-Pichel, and Stout [30]. Samples were processed at 95 °C for 3 min, followed by 39 cycles at 95 °C for 10 s and 55 °C for 30 s. The reference gene was Npun_R0035, which encodes for DNA gyrase, a housekeeping protein that has been used in similar studies [30,31,36]. Expression levels were normalized to those of the reference gene and the fold change between stressed and unstressed cells for all of the treatments was determined using the ΔΔCq formula for calculating normalized expression [37] in the CFX Manager software (Bio-Rad Laboratories). Statistical comparisons were conducted with triplicate biological replicate data using a student’s *t*-test between the treated and untreated samples, with a *p*-value of <0.05 regarded as significant.

## 3. Results

### 3.1. Scytonemin Production

**Figure 2:**
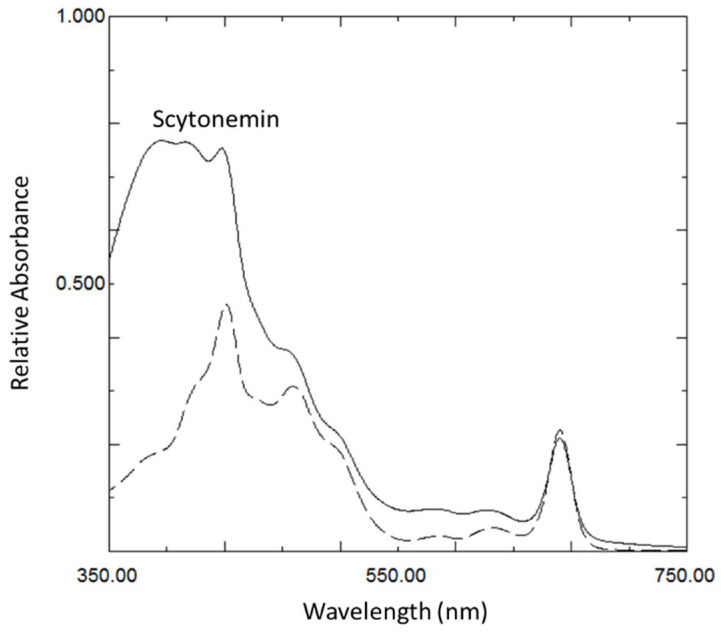
Representative absorption spectra of lipid-soluble pigments extracted from the cyanobacterium *Nostoc punctiforme* after exposure to UVA for 48 h with scytonemin (solid line) compared to high light for 48 h without scytonemin (dashed line). The strong absorption at 384 nm is indicative of scytonemin. Similar results were obtained for UVB (similar to the solid line) as well as high light and osmotic stress (similar to the dashed line).

Scytonemin was extracted from the stressed cells to determine the relationship between scytonemin production and the expression of scytonemin biosynthetic genes. In response to 48 h of UVA and UVB exposure, scytonemin was produced by the cells (Figure 2). However, scytonemin was not produced in response to high light and osmotic stress using the same duration of exposure.

### 3.2. Gene Expression

Experiments were conducted separately for each condition, with dedicated control experiments conducted in parallel for each condition. The gene expression analysis revealed the transcript abundance of scytonemin genes *scyA*, *trpB*, and *eboE* in response to each condition. Both *scyA* and *trpB* were upregulated in all of the conditions except for osmotic stress compared to the unstressed cells (Table 1). Interestingly, *trpB* had a stronger response for UVA, while *scyA* was stronger for UVB and high light stress. Osmotic-stressed samples showed a downregulation of *scyA* and *trpB* in comparison to the unstressed samples. The *eboE* gene expression response was more variable. There were no significant changes in gene regulation for *eboE* in response to UVA compared to the untreated samples. However, in cells treated with UVB and osmotic stress, *eboE* was downregulated, and for those exposed to high light, it was upregulated.

## 4. Discussion

Scytonemin is a highly effective molecule that plays an essential role in defending cyanobacteria against the harmful consequences of UVA exposure. It was hypothesized that the genes associated with sunscreen biosynthesis would be expressed in response to other environmental conditions. This was in part based on the broad ecological distribution of scytonemin-producing strains, as well as the previous literature [12,28]. The results mostly support the hypothesis by the upregulation of *scyA* and *trpB* genes in response to UVA, UVB, and high light. In response to osmotic stress, there was a downregulation in *scyA* and *trpB*. In a previous study, osmotic stress in conjunction with UVA showed an inhibitory effect on scytonemin production [28]. Although the previous study did not include a transcriptional abundance parameter, it is reasonable to deduce that scytonemin genes were not upregulated. 

The *ebo* gene cluster was studied since it is related to scytonemin biosynthesis, specifically to the export of scytonemin precursors across the periplasm [24]. The *eboE* gene showed a moderate upregulation when exposed to high light and a moderate downregulation under UVB, but overall showed a greater decrease in fold change reaction as compared to *scyA* and *trpB*. This result is consistent with a previous study that showed that the expression of all the *ebo* genes followed the peak in the expression of the biosynthetic genes [30]. This temporal distinction between the biosynthetic genes and the *ebo* genes suggests that they play a post-biosynthetic role in the production of scytonemin, such as secretion or post-translation modification. Therefore, the role of the *ebo* cluster in export could explain the delayed gene expression response.

Although there was upregulation of the scytonemin genes in cultures treated with high light, scytonemin was not produced, in contrast to UVA and UVB stress. The differences in scytonemin production when comparing UVA, UVB, and high light suggest that other factors play a role in pigment production. One possibility is that the proteins involved in scytonemin biosynthesis are subject to post-translational modification and that high light does not regulate this process. While the post-translational regulatory mechanisms of these proteins are unknown, ScyF (Npun_R1271) contains a NHL domain subgroup (IPR013017) that could be involved in protein–protein interactions [27]. It is possible that this protein–protein interaction or others rely on post-translational modification. Alternatively, additional genes in the *N. punctiforme* genome could be involved in scytonemin biosynthesis, which are not expressed under high light. Still another possibility for the lack of pigment production under high light could be a time-dependent response for scytonemin production. The specific time point that transcripts and end products are measured is critical to the interpretation of the data, since transcript levels change rapidly and product synthesis can lag for days. In fact, scytonemin production has been shown to occur in response to high light over 99 µmols photons m^−2^ s^−1^ after 4 days of exposure, especially with the addition of blue light [12]. Therefore, the failure to detect scytonemin could be due to the fact that the stress period of 2 days was not sufficient for translation and enzyme activity to occur. Time was also shown to be a factor in a temporal study of expression that showed expression of the scytonemin biosynthetic genes after 48 h of UVA stress, while the *ebo* genes were not upregulated until 96 h [30].

## 5. Conclusions

The light-associated conditions effectively induced the expression of scytonemin genes, while osmotic stress did not. While this was expected, it was also surprising since scytonemin was not produced under high light stress. This can possibly be explained by unknown post-translational modifications or additional genes necessary for pigment production, which are not induced by high light. Alternatively, it could simply be due to differences in the temporal response between expression and biosynthesis. Regardless of the specific reason that high light did not result in pigment production, several possibilities exist that could be explored in future studies. These results emphasize the significance of studies targeting the intersection of the physiological response and specific gene expression response, rather than inferring this relationship from other studies. Overall, our results support previous studies, and based on the expression of genes under high light that did not result in pigment production, the data also suggest that additional mechanisms may be involved in the biosynthesis and regulation of this pigment.

## Figures and Tables

**Table 1:** Pigment production and transcript abundance of *scyA, trpB*, and *eboE* following 48 h of stress.

Stress	Scytonemin	Gene	Fold Change	*p*-Value
UVA	Yes	*scyA*	+26.05	0.049 *
		*trpB*	+77.53	0.039 *
		*eboE*	−1.63	0.278
UVB	Yes	*scyA*	+22.7	0.002 *
		*trpB*	+2.23	0.001 *
		*eboE*	−3.01	0.001 *
High Light	No	*scyA*	+28.59	<0.001 *
		*trpB*	+4.63	<0.001 *
		*eboE*	+3.59	<0.001 *
Osmotic	No	*scyA*	−4.90	0.002 *
		*trpB*	−3.12	0.001 *
		*eboE*	−1.53	0.010 *

* *p* < 0.05 indicates a significant difference in gene expression between triplicate treated and untreated cultures.

## Data Availability

The data presented in this study is available at https://users.pfw.edu/soulet/pubs.html (accessed on 23 December 2021).
